# A Case of Monochorionic-Triamniotic Triplet Pregnancy with TRAP Sequence Successfully Treated with Radiofrequency Ablation with a Parallel Circuit Consisting of Anastomosed Blood Vessels between the Direct Pump Fetus and the Indirect Pump Fetus

**DOI:** 10.1155/2019/5319028

**Published:** 2019-03-25

**Authors:** Shusaku Kobori, Masatake Toshimitsu, Shinichi Nagaoka, Jun Murotsuki

**Affiliations:** Department of Maternal and Fetal Medicine, Miyagi Children's Hospital, Sendai, Miyagi 989-3126, Japan

## Abstract

Monochorionic-triamniotic triplet pregnancy with twin reversed arterial perfusion (TRAP) sequence is one of the rare complications of multiple pregnancy and has been reported by only a few. Here, we report a case of monochorionic-triamniotic triplet pregnancy with TRAP sequence successfully treated with radiofrequency ablation, which did not develop polyhydramnios and heart failure although the estimated weight of the acardiac fetus increased twice as much as that of the direct pump fetus. Interestingly, the anastomosed blood vessels between the direct and indirect pump fetuses comprised a parallel circuit, which provided blood flow to the acardiac fetus. We hypothesized that the burden on the pump fetus in monochorionic pregnancy with TRAP sequence would be different between triplet and twin pregnancies.

## 1. Introduction

Twin reversed arterial perfusion (TRAP) sequence is one of the rare complications of multiple pregnancy. It occurs for approximately 1% of monochorionic pregnancies [1]. Monochorionic triplet pregnancies occur at a rate of 1 in 45000 deliveries [2]. However, with progress of assisted reproductive technologies, the rate of multiple pregnancies (triplets and above) has dramatically risen over the last 20 years. Accordingly, the rate of monochorionic tripletpregnancy with TRAP sequence seems to have increased during the same period. In monochorionic twin pregnancy with TRAP sequence, there are several vascular anastomoses between an acardiac fetus and the other fetuses. In monochorionic triplet pregnancy with TRAP sequence, although there are several vascular anastomoses between an acardiac fetus and one fetus, the remaining 3rd fetus could also send blood indirectly via another vascular anastomoses. We hypothesize that the burden on the pump fetus of monochorionic triplet pregnancy with TRAP sequence would be different from that of monochorionic twin pregnancy with TRAP sequence. We report a case of monochorionic-triamniotic triplet pregnancy with TRAP sequence successfully treated with radiofrequency ablation, which did not develop polyhydramnios and heart failure although the estimated weight of the acardiac fetus increased twice as much as that of the direct pump fetus.

## 2. Case

A 38-year-old woman, gravida 4 para 1, was referred due to monochorionic-triamniotic triplet pregnancy at 10 weeks of gestation. We confirmed monochorionic-triamniotic triplet gestation, with absence of cardiac activity in one triplet. Reversed flow in the fetal umbilical cord was demonstrated on Doppler ultrasound examination. These findings were consistent with monochorionic-triamniotic gestation complicated with twin reversed arterial perfusion (TRAP) sequence. The acardiac fetus did not have an upper body.

Using Doppler ultrasound, the presence of anastomotic vessels was confirmed between triplets A (acardiac fetus) and B (direct pump fetus) but not between triplet A and triplet C (indirect pump fetus). The Doppler measurements of feeding vessel (umbilical artery) in the acardiac twin revealed pulse rate that was similar to FHR of twin B. The direct pump fetus could be distinguished from the indirect pump fetus by measuring FHR. The estimated fetal body weight (EFBW) of the acardiac fetus increased as pregnancy progressed. EFBW of an acardiac fetus can be obtained by the following equation: (grams) = (-1.66 × longest length [cm]) + (1.21 × longest length [cm]^2^) [3]. At 14 weeks, body weight was 136 g for triplet A, 56 g for triplet B, and 59 g for triplet C. At 16 weeks, body weight was 227 g for triplet A, 109 g for triplet B, and 120 g for triplet C. In triplets* B* and* C*, we could not confirm polyhydramnios, absent umbilical artery end-diastolic velocity, umbilical venous pulsation, and absent or reverse blood flow in the ductus venosus.

**Figure 1:**
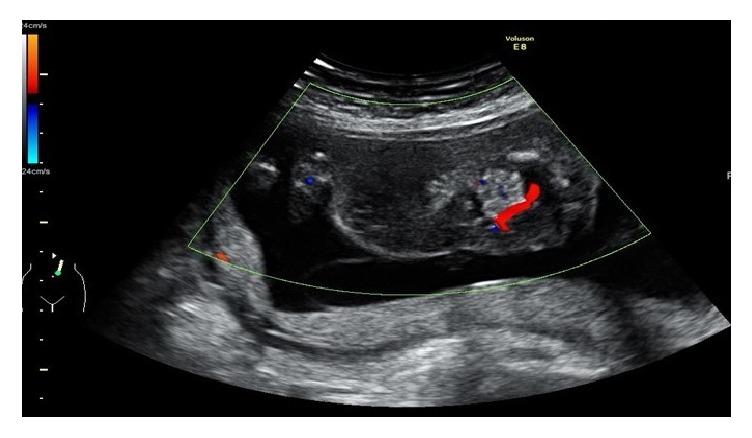
Ultrasonic color Doppler image of the acardiac fetus at 16 weeks of gestation. Blood flow is observed in the navel of the acardiac fetus, which is stuck to the anterior uterine wall. Complete cessation of blood flow in the acardiac fetus was successful with a single puncture.

We usually perform RFA when EFBW of an acardiac fetus is 0.7 times or more as much as that of a pump fetus, and heart failure and amniotic fluid excess are recognized. In the current case, weight of the acardiac fetus exceeded twice that of the pump fetus and weight of the acardiac fetus had increased further. We speculated that the pump fetus would develop heart failure with high probability in the future; thus, radiofrequency ablation (RFA) was performed at 18 weeks. The acardiac fetus was stuck to the anterior uterine wall and the other sac did not interfere with needle insertion (Figure 1). We used a 480 kHz radiofrequency generator and 20 cm-long, 17-gauge, internally cooled electrode with a 2 cm-long exposed metallic tip (Cool-tip RF system; Valleylab, Boulder, Co., USA). The patient had strong anxiety in a conscious state, so we performed surgery with general anesthesia. An electrode was transabdominally introduced and inserted into the fetal abdomen adjacent to the umbilical cord insertion area under continuous ultrasound guidance. Coagulation was initiated for 90 seconds, and complete blood flow cessation was successful in the acardiac fetus. In triplets B and C, preoperative and postoperative middle cerebral artery peak systolic velocities were the same and fetal anemia was not observed (Table 1).

**Figure 2:**
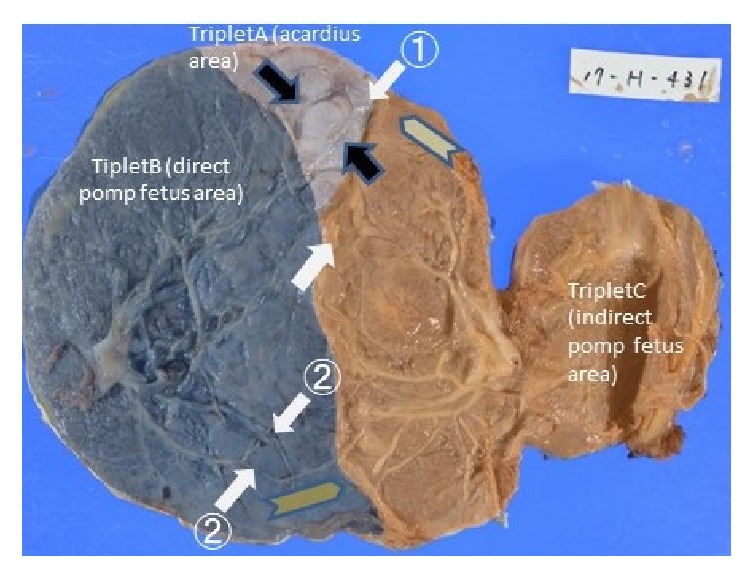
Photograph of the placental vascular surface. The black arrow indicates the anastomoses between the acardiac fetus and the direct pump fetus. The white arrow indicates the anastomoses between the direct pump fetus and the indirect pump fetus. ① is the anastomoses between the umbilical artery of the direct pump fetus and the umbilical artery of the indirect pump fetus. ② is the anastomoses between the umbilical vein of the direct pump fetus and the two umbilical veins of the indirect pump fetus. The gray arrow indicates the direction of blood flow in the vascular anastomoses.

During follow-up by weekly ultrasound, twin-to-twin transfusion syndrome, selective intrauterine growth restriction, and twin anemia–polycythemia sequence did not develop. At 37 weeks, a cesarean section was performed; two male newborn infants were delivered. Triplet B weighed 2,662 g, with Apgar scores of 8 and 9 at 1 and 5 minutes, respectively. Triplet C weighed 2,031 g, with Apgar scores of 8 and 9 at 1 and 5 minutes, respectively. Placental examination showed four vascular anastomoses between triplets B and C (Figure 2) and vascular anastomoses (i.e., umbilical artery and umbilical vein) between triplets A and B. The umbilical artery between triplets A and B was anastomosed with a thin umbilical artery of triplet C. The umbilical vein between triplets A and B was anastomosed with two umbilical veins of triplet C. At 12 months, both children showed normal neurodevelopment.

## 3. Discussion

This is the first report on two liveborn infants delivered in term after successful RFA for monochorionic-triamniotic triplet pregnancy with TRAP sequence. There were two similar reports; however, the liveborn infants were preterm [4, 5]. In the current case, we succeeded in complete blood flow cessation with a single puncture on the acardiac fetus stuck to the anterior uterine wall.

The treatment methods of TRAP sequence are mainly RFA, bipolar cord coagulation, and intrafetal (or interstitial) laser coagulation. Recently, RFA and intrafetal (or interstitial) laser coagulation are mainly reported [6–8]. Laser surgery is generally selected at 14–16 weeks, and RFA is selected at 16–18 weeks because of fewer rate of premature labor [7]. Neonatal survival rate was comparable between the RFA and laser techniques (85%* vs* 82%,* P* = 0.63) [7]. In the current case, we decided intrauterine operation at 18 weeks, so we performed RFA.

Lee et al. [6] reported that there were no maternal deaths and no women required transfusions in patients treated for RFA. However, two women suffered thermal injuries at the site of the grounding pads. As a remedy, they changed to bigger grounding pads and had no further maternal thermal injuries. The cool-tip RF system we used this time comprised adopted pads which are bigger than the ground pads used at that time, and no complication was admitted.

Vascular anastomoses between triplets A and B were confirmed by Doppler ultrasound examination. Takahashi et al. reported that pump fetus identification was possible using dual-gate Doppler (ARIETTA 70) [5]. Here, we could distinguish the pump fetus from the indirect pump fetus based on the different baseline FHRs, which had relatively few fluctuations during gestation.

In monochorionic twin pregnancy with TRAP sequence, an acardiac fetus with an EFBW ≥0.7 times greater than that of the pump fetus is likely to develop heart failure [3]. Here, there was no abnormal blood flow or any sign of heart failure in triplet B (direct pump fetus), although the estimated weight of triplet A increased twice as much as that of triplet B. It can be inferred that triplet A was supplied with blood flow from triplet B and triplet C (indirect pump fetus) via another vascular anastomoses; this likely prevented triplet B from developing heart failure.

Without in utero treatment, the perinatal mortality rate for pump twins was approximately 55% [3].Lewi et al. reported that intrauterine fetal death (IUFD) rate was the highest between the first and second trimester. It is also said that a poor prognostic factor of IUFD is a sudden increase in a pump fetus and accompanying heart failure [8]. In the current case, the direct pump fetus and the indirect pump fetus did not develop heart failure. However, the total weight of the direct pump fetus and the indirect pump fetus was almost the same as the acardiac fetus, and it was judged that the direct pump fetus and the indirect pump fetus are likely to develop heart failure in the future, and RFA was performed. It was possible to perform RFA before the onset of heart failure, leading to improvement in the prognosis of the both fetuses.

**Figure 3:**
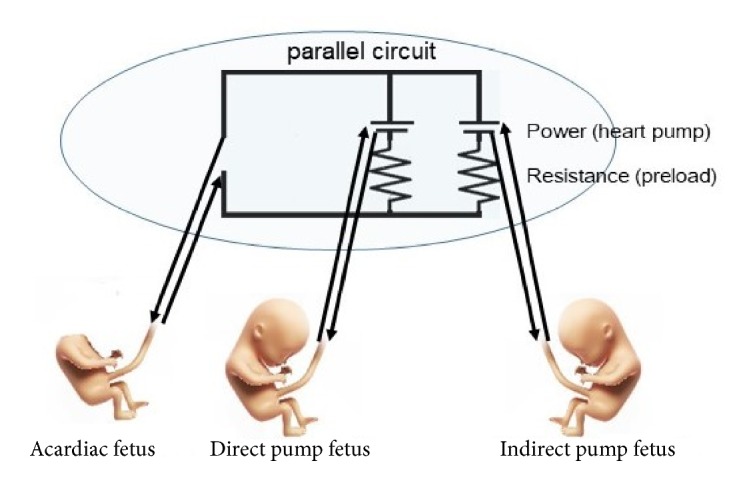
Schematic representation of the anastomosed blood vessels between triplets B and C. The anastomosed blood vessels comprised a parallel circuit, which provided blood flow to triplet A.

Argoti et al. [4] reported that blood flow to the acardiac fetus would be from direct pump and indirect pump fetuses via anastomosed blood vessels. However, the mechanism was not well understood. In their case, two live fetuses were simultaneously diagnosed with polyhydramnios; however, they did not develop heart failure. The fact that both direct pump and indirect pump fetuses simultaneously developed polyhydramnios indicated that the acardiac fetus was supplied with blood flow from both fetuses. Here, the umbilical artery between triplets A and B was anastomosed with a thin umbilical artery of triplet C. The umbilical vein between triplets A and B was anastomosed with two umbilical veins of triplet C.  Figure 3 shows that the anastomosed blood vessels between triplets B and C comprised a parallel circuit, which provided blood flow to triplet A.

We hypothesized that increased preload has increased vascular resistance in triplet B. When vascular resistance of triplet B became higher than that of triplet C, blood flow from the acardiac fetus was probably diverted from triplet B to triplet C. In the previously reported cases, simultaneous polyhydramnios development in direct pump and indirect pump fetuses was probably because their preloads were equal. In an ideal parallel circuit, workloads of triplets B and C can be equal. However, there may have been other factors, such as placenta, blood flow, and preload, which differed between triplets B and C. Nevertheless, the burden on the pump fetus in monochorionic pregnancy with TRAP sequence can be lighter in triplet pregnancy than in twin pregnancy.

In the previous report [4], the capacity load of the direct pump fetus and the indirect pump fetus slowly progressed although the acardiac fetus rapidly increased. They succeeded in performing RFA before developing heart failure. From this and the previous cases, it appears that the progress of monochorionic-triamniotic triplet pregnancy with TRAP sequence is slow; it may be a disease in which it is possible to perform intrauterine therapy at the most appropriate timing before prognosis is poor. Therefore, monochorionic-triamniotic triplet pregnancy with TRAP sequence should be offered as an option of intrauterine therapy, such as RFA, to improve neonatal survival rate.

## Figures and Tables

**Table 1:** Blood flow changes of the direct pump fetus and the indirect pump fetus around RFA.

	Direct pump fetus (fetus B)		Indirect pump fetus (fetus C)	
	Pre-intervention	Post-intervention	Pre-intervention	Post-intervention
UA-PI	1.25	1.19	0.97	0.95
MCA-PSV (cm/s)	24.3	22.2	21.5	23.4
DV-PI	0.42	0.48	0.37	0.45
UV-pulsation	no	no	no	no
MVP (cm)	4.3	4.5	3.8	4

UA-PI, pulsatility index of the umbilical artery; MCA-PSV, middle cerebral arterial peak systolic velocity; DV-PI, pulsatility index of the ductus venosus; UV-pulsation, pulsation of the umbilical vein; MVP, maximal vertical pocket.
